# Cutaneous kinase activity correlates with treatment outcomes following PI3K delta inhibition in mice with experimental pemphigoid diseases

**DOI:** 10.3389/fimmu.2022.865241

**Published:** 2022-09-28

**Authors:** Saeedeh Ghorbanalipoor, Shirin Emtenani, Melissa Parker, Mayumi Kamaguchi, Colin Osterloh, Manuela Pigors, Natalie Gross, Stanislav Khil’chenko, Anika Kasprick, Sabrina Patzelt, Diana Wortmann, Ibrahim O. Ibrahim, Kentaro Izumi, Stephanie Goletz, Katharina Boch, Kathrin Kalies, Katja Bieber, Paul Smith, Enno Schmidt, Ralf J. Ludwig

**Affiliations:** ^1^ Lübeck Institute of Experimental Dermatology and Center for Research on Inflammation of the Skin, University of Lübeck, Lübeck, Germany; ^2^ Incyte Research Institute, Wilmington, DE, United States; ^3^ Department of Dermatology, University of Lübeck, Lübeck, Germany; ^4^ Institute of Anatomy, University of Lübeck, Lübeck, Germany

**Keywords:** animal model, neutrophils, autoimmunity, pemphigoid, epidermolysis bullosa acquisita, mucous membrane pemphigoid, signal transduction, phosphatidylinositol 3-kinase (P13k)

## Abstract

Chronic blistering at the skin and/or mucous membranes, accompanied by a varying degree of inflammation, is the clinical hallmark of pemphigoid diseases that impose a major medical burden. Pemphigoid diseases are caused by autoantibodies targeting structural proteins of the epithelial basement membrane. One major pathogenic pathway of skin blistering and inflammation is activation of myeloid cells following Fc gamma receptor-dependent binding to the skin-bound immune complexes. This process requires activation of specific kinases, such as PI3Kδ, which have emerged as potential targets for the treatment of pemphigoid diseases. Yet, it is unknown if global cutaneous kinase activity present in lesional pemphigoid disease correlates with therapeutic effects following treatment with a given target-selective kinase inhibitor. To address this, we here first determined the kinase activity in three different mouse models of pemphigoid diseases: Antibody transfer-induced mucous membrane pemphigoid (MMP), antibody transfer-induced epidermolysis bullosa acquisita (EBA) and immunization-induced EBA. Interestingly, the kinome signatures were different among the three models. More specifically, PI3Kδ was within the kinome activation network of antibody transfer-induced MMP and immunization-induced EBA, but not in antibody transfer-induced EBA. Next, the therapeutic impact of the PI3Kδ-selective inhibitor parsaclisib was evaluated in the three model systems. In line with the kinome signatures, parsaclisib had therapeutic effects in antibody transfer-induced MMP and immunization-induced EBA, but not in autoantibody-induced EBA. In conclusion, kinase activation signatures of inflamed skin, herein exemplified by pemphigoid diseases, correlate with the therapeutic outcomes following kinase inhibition, demonstrated here by the PI3Kδ inhibitor parsaclisib.

## Introduction

Pemphigoid diseases are prototypical organ-specific autoimmune diseases of the skin and/or mucous membranes that are characterized and caused by autoantibodies targeting structural proteins of the basement membrane of surface epithelial cells. Depending on the clinical presentation and the targeted autoantigens, distinct pemphigoid diseases are diagnosed (1, 2). These include mucous membrane pemphigoid (MMP) and epidermolysis bullosa acquisita (EBA). Both MMP and EBA are difficult to treat and mainly rely on immunosuppressive therapy (3–7). Novel treatment options are urgently needed, whereby the phosphatidylinositol-3-kinase (PI3K) pathway represents a promising target for the treatment of autoimmune blistering diseases (8, 9).

In pemphigoid disease, once pathogenic autoantibodies are generated (for example, BP180 and/or and laminin 332 in MMP, and type VII collagen (COL7) in EBA), they bind to their respective target antigen(s) expressed in the skin and/or mucosa (10). Autoantibody binding triggers subepidermal blistering and inflammation through recruitment of polymorphonuclear leukocytes (PMNs) into the skin. Here, PMNs bind to the immune complexes located at the anchoring junctions between the epithelium and the extracellular matrix in skin and mucosal tissue *via* their activating Fc gamma receptors (FcγRs). This triggers an intracellular signaling cascade, leading to the release of reactive oxygen species (ROS) and proteases that ultimately cause subepidermal blistering and inflammation (11–13).

With the approval of (topical) kinase inhibitor treatments in other chronic inflammatory skin diseases, such as phosphodiesterase 4- or Janus kinase (JAK) inhibitors for the treatment of atopic dermatitis (14, 15), signaling events in PMNs following FcγR-mediated binding to the tissue-bound immune complexes have emerged as potential pharmacological targets for the treatment of pemphigoid diseases (16–18). Among others, PI3Kβ and δ are involved in immune complex-induced PMN activation and their inhibition was shown to reduce disease severity in pemphigoid disease mouse models (19, 20).

However, the vast majority of research on cell signaling in pemphigoid diseases has been hypothesis-driven so far, and centered on the events following the FcγR-dependent binding of PMNs to immune complexes (21–25). Thus, an unbiased and complete overview of kinase activation signatures in pemphigoid diseases is missing. In addition, it is also unknown if a specific kinase activation profile in inflamed skin is associated with the treatment outcomes following kinase inhibition. To address these knowledge gaps, we systematically evaluated the kinase activation signatures in three different mouse models of pemphigoid diseases: Antibody transfer-induced MMP, antibody transfer-induced EBA and immunization-induced EBA (26). In parallel, we evaluated the impact of PI3Kδ inhibition using parsaclisib, a PI3Kδ-selective inhibitor in late clinical development, on clinical disease manifestation in the three different pemphigoid disease models. Ultimately, we investigated if the kinome signature of each disease model correlates with the observed treatment outcomes.

## Materials and methods

### Experiments with human biomaterials

Skin and blood collection from healthy volunteers or patients was performed after written informed consent was obtained. All experiments with human samples were approved by the ethical committee of the Medical Faculty of the University of Lübeck and performed in accordance with the Declaration of Helsinki.

### Mice

C57BL/6J (B6) and B6.SJL-H2^s^ C3^c^/1CyJ (B6.s) mice were obtained from colonies held at the animal facility at the University of Lübeck. Animals were fed acidified drinking water and standard chow ad libitum and were held on a 12-hour light-dark cycle. Sex-matched mice aged 6-10 weeks were used for the experiments. All experiments were approved by the Animal Care and Use Committee (Kiel, Germany) and performed by certified personnel. All clinical examinations, biopsies, and bleedings were performed under anesthesia using i.p. administration of a mixture of ketamine (100 mg/g, Sigma-Aldrich) and xylazine (15 mg/g, Sigma-Aldrich). The percentage of the affected body surface area was determined in a blinded manner.

### Chemicals

Parsaclisib (INCB050465), a potent, selective, and orally active inhibitor of PI3Kδ, was synthesized as described (27). For *in vitro* studies, the drug was initially diluted in 100% DMSO (Sigma-Aldrich, Hamburg, Germany). Of this, different concentrations (500, 200, 20, 5, 2.5, 1, 0.5, and 0.1 nM) were prepared in 0.1% DMSO. For *in vivo* administration 0.3, 1, or 3, mg/kg of parsaclisib was freshly prepared in 95% (v/v) of methylcellulose solution (0.5% (w/v)) and 5% (v/v) N,N-Dimethylacetamide (DMA; Sigma-Aldrich). Parsaclisib was dosed twice daily by oral gavage. Methylprednisolone (MP) (Urbason™) was purchased from Sanofi-Aventis (Frankfurt, Germany). MP was used as a reference treatment and injected intraperitoneally (i.p.) once daily at a concentration of 20 mg/kg.

### PMNs’ reactive oxygen species release assay upon stimulation with immune complexes

Immobilized immune complexes were formed using recombinant proteins of h-COL7EF and anti-h-COL7 IgG1, as previously described (28). In brief, a 96-well white plate (Greiner BioOne, Frickenhausen, Germany) was coated with 10 µg/mL of h-COL7EF in 50 mM carbonate/bicarbonate buffer (pH 9.6) for 3 h. Afterwards, wells were blocked and subsequently incubated for 2 h with 2 μg/mL of anti-h-COL7 IgG1 or PBS. In parallel, PMNs were isolated from healthy donors using PolymorphPrep™ (Axis-Shield GmbH, Heidelberg, Germany) according to the manufacturer’s instructions. After purification, PMNs were resuspended in chemiluminescence medium (color-free RPMI-1640 (Millipore Sigma) supplemented with 1% heat-inactivated fetal calf serum (FCS), 2 g/l glucose, and 25 mM HEPES) containing 1 mM luminol (5-amino-2,3-dihydro-1,4-phthalazindione; Sigma-Aldrich). 2×10^5^ PMNs were seeded into each well. In case of murine PMNs, bone marrow cells were harvested from C57BL/6J mice by flushing femurs and tibias with cold HBSS buffer supplemented with 0.5% FCS and 20 mM HEPES. Cells were collected by centrifugation and filtered through a 70 μm strainer. Neutrophils were isolated from the cell suspension using the Neutrophil Isolation Kit, mouse, an LS Column, and a MidiMACS Separator (Miltenyi Biotec GmbH, Teterow, Germany) following the manufacturer’s protocol. A total of 2×10^5^ PMNs were added to each well in the presence of 0.1% DMSO or parsaclisib (at the concentrations of 500, 200, 20, 5, 2.5, 1, 0.5, and 0.1 nM). 10 ng/mL of phorbol-myristate-acetate (PMA; Sigma-Aldrich) served as positive control. The chemiluminescence reaction was analyzed at 37°C for 60 repeats using a GloMax^®^ Discover System microplate reader (Promega GmbH, Mannheim, Germany).

### Cell-free ROS release assay

To exclude that parsaclisib acts as a ROS scavenger, a cell-free ROS release assay was employed as previously reported (29). In brief, human myeloperoxidase (MPO; Enzo Life Sciences, Lörrach, Germany), catalase (CAT; Sigma-Aldrich), and glucose oxidase (GOX; Sigma-Aldrich) were mixed at final concentrations of 10 mU/mL, 2 U/mL, and 0.5 U/mL, respectively. The mixed substances were added to HBSS buffer containing 0.1% v/v Triton X-100, 5 mM glucose, and 2 mM luminol. Subsequently, the medium was supplemented with 0.1% DMSO or parsaclisib at concentrations of 500, 200, 20, 5, 2.5, 1, 0.5, and 0.1 nM. 10 mM N-acetyl-L-cysteine (NAC; Sigma-Aldrich) was used as a positive control. ROS generation was monitored as stated above.

### Flow cytometric cytotoxicity assay

In order to evaluate a potential drug-induced toxicity, a flow cytometric assay was used: PMNs, isolated from healthy donors by PolymorphPrep™, were seeded into the plate and incubated with parsaclisib (500, 200, 20, 5, 2.5, 1, 0.5, and 0.1 nM) for 2 h at 37°C. After centrifugation, the pellet was resuspended in Annexin V Binding Buffer (BioLegend GmbH, Fell, Germany). FITC Annexin V (BioLegend GmbH) was then added to the cell suspension at a final concentration of 1 μg/mL. Following 20 min incubation at 4°C, cells were washed, and the pellet was resuspended in PBS with 1% BSA. Propidium iodide (PI; Miltenyi Biotec GmbH) was added to the samples (dilution, 1:300) and cytotoxicity was measured using a MACSQuant^®^ Analyzer 10 (Miltenyi Biotec GmbH).

### *Ex vivo* dermal-epidermal separation (cryosection) assay

Following published protocols (30, 31), cryosections of human skin, that was obtained from elective plastic surgery, were incubated with immunoapheresis materials or IgG isolated from serum of two EBA patients using protein G columns, of EBA patients for 1 h at 37°C, followed by addition of human PMNs isolated by dextran sedimentation (Carl Roth, Karlsruhe, Germany) of freshly collected, heparinized blood from healthy volunteers (n=3). Afterwards, 0.5 ml of PMNs (2-2.5 × 107 cells), pre-incubated with solvent or parsaclisib at 2, 20, and 200 nM. PMNs, pre-incubated with solvent or parsaclisib at varying concentrations (2, 20, and 200 nM/mL) for 15 min at 37°C, were added onto the skin sections. Slides were further incubated at 37°C for 3 h. After washing, slides were fixed in formalin and H&E-stained. Using ImageJ software (https://imagej.nih.gov/ij/), DES split formation was quantified. The percentage of dermal-epidermal separation (DES) was calculated as length of separation divided by total length of the dermal-epidermal junction (DEJ) on skin section measured on a Keyence microscope (BZ-9000 series, Keyence GmbH, Neu-Isenburg, Germany).

### Chemotaxis assay

Human PMNs were freshly isolated from healthy donors using a standard Ficoll-PaqueTM Plus (GE Healthcare Europe GmbH, Freiburg, Germany) density gradient method. The effect of parsaclisib on PMN chemotaxis was assessed by Boyden chamber (20). In brief, interleukin (IL)-8 (6 nM; Peprotech, Hamburg, Germany) was diluted in PBS with Ca^2+^, Mg^2+^/0.1% BSA and added to the bottom wells of the chamber. After covering the bottom wells with a polycarbonate membrane (pore size: 5 µm; Costar Nucleopore GmbH, Tübingen, Germany), the top wells were filled with freshly prepared PMNs (1 × 10^5^ cells/well) in PBS with Ca^2+^, Mg^2+^/1% BSA that were pre-incubated for 10 min at 37°C with parsaclisib (500, 200, 20, 5, 2.5, 1, 0.5, and 0.1 nM). After 1-hour incubation at 37°C, the chamber was disassembled and migrated cells were transferred from the bottom wells to a microtiter plate. Afterwards, migrated neutrophils were lysed by 0.1% (w/v) hexadecyltrimethylammonium bromide solution (Millipore Sigma). Number of migrated neutrophils was determined *via* endogenous MPO activity by adding TMB substrate solution (Thermo Fisher Scientific, Dreieich, Germany). The redox reaction was stopped by 1 M H_2_SO_4_ and oxidized tetramethylbenzidine was measured at OD450 nm using a GloMax^®^ microplate reader. The number of migrated neutrophils was calculated from a standard curve of cell lysates run in parallel.

### Pre-clinical models of pemphigoid diseases

#### Antibody transfer-induced EBA

Induction of experimental EBA by antibody transfer in adult B6 mice was performed as described (26). B6 mice were selected for these experiments because they are among the most susceptible strains for antibody transfer-induced EBA (32, 33). Administration of MP, solvent or parsaclisib (0.3, 1, or 3 mg/kg) was initiated on day 0 of the experiment and continued until day 11. For randomization, mice (n=1 per randomization cycle) were allocated to each treatment on an alternating basis, independent of cages. Specifically, the first, 6^th^, 11^th^, … mouse was allocated to MP, the 2^nd^, 7^th^, 12^th^, … to solvent, and so forth. Solvent and parsaclisib were applied by oral gavage, while MP was applied i.p. To score the severity of disease, skin areas exhibiting erythema, blisters, erosions, crusts, or alopecia were determined by an observer unaware of the applied treatments on days 4, 8 and 12 as described in detail elsewhere (26). Blood and tissue samples were collected on day 12.

#### Immunization-induced EBA

Following established protocols (26), B6.s mice were immunized with 120 μg of vWFA2 domain of mCOL7 (mCOL7^vWFA2^) emulsified (1:1) in Titermax^®^ (CytRx Corporation, CA, USA). B6.s mice were selected for these experiments because they are one of the few susceptible strains for immunization-induced EBA (34, 35). When 2% or more of the body surface area was affected by EBA skin lesions in an individual animal, this mouse was randomly allocated into the following groups: solvent, MP or parsaclisib (0.3, 1 or 3 mg/kg). Randomization was performed as described for antibody transfer-induced EBA. Again, solvent and parsaclisib were applied by oral gavage, while MP was applied i.p. Clinical scoring was performed once per week by an observer unaware of the applied treatments. Relative clinical scores were calculated by normalizing the weekly score to the initial clinical score when allocated into treatment groups. Treatments were carried out for a total period of 4 weeks. Serum samples were obtained weekly for determination of circulating total and antigen-specific IgG.

#### Antibody-transfer induced MMP

To generate IgG against the murine α3 chain of laminin 332 (mLAMα3), New Zealand white rabbits were immunized with a mixture of recombinant His-tagged fragments of middle and C-terminal mLAMα3 portions (11). Total rabbit IgG was affinity purified using protein G Sepharose (Genscript, Piscataway, NJ, USA). Reactivity of purified IgG was analyzed by indirect immunofluorescence (IF) microscopy on murine skin. B6 mice were injected every other day with 6 mg of rabbit anti-mLAMα3 IgG or normal rabbit (NR) IgG. B6 mice were selected for these experiments because they are so far the only strain used in this model (11). Randomization and treatments were performed as described for antibody transfer-induced EBA. Different body parts were individually scored by the appearance of crust, erythema, lesions, and alopecia. On Day 12, high-resolution endoscopy (HOPKINS Optik 64019BA; Karl StorzAidaVet, Tuttlingen, Germany) of mouth oral cavity (i.e., pharyngeal mucosa, tongue, and right/left buccal) was conducted to determine the extent of oral lesions. Blood and tissue samples were taken for further analysis. For oral score, we determined 1 score point for each affected area: tongue, left buccal mucosa, right buccal mucosa, pharynx.

### Multiplex kinase activity profiling

Kinase activity profiles were determined using PamChip^®^ 4 microarray system (PamGene International B.V., ‘s-Hertogenbosch, The Netherlands) (36). Thirty 10 μm-thick frozen slices from lesional ear biopsies were cut and lysed using M-PER (Mammalian Protein Extraction Reagent; Thermo Fisher Scientific) containing 1% (v/v) Protease and Phosphatase Inhibitor Cocktail (Thermo Fisher Scientific). After centrifugation (15 min, 4°C, 10,000xg), the supernatants were snap frozen and stored at -80°C. Protein concentration was determined using BCA assay kit (Thermo Fischer Scientific) according to the manufacturer’s instructions. The Serine-Threonine Kinase (STK) and Protein Tyrosine Kinase (PTK) microarray assays were performed according to the manufacturer’s instructions. A FITC-conjugated anti-phosphotyrosine antibody was used for visualization during and after the pumping of lysates through the three-dimensional surface of the array. The capture of substrate phosphorylation signals was enabled by a computer-controlled CCD camera and measured repeatedly during a 1-hour kinetic protocol using the Evolve software (PamGene International B.V.). The analysis of the images was performed using the Bionavigator Software (Ver. 6.3). A kinase was considered to be modulated (either activated or inhibited) if it had a mean specificity score (negative decadic logarithm of the likelihood of obtaining a higher difference between the groups when assigning peptides to kinases randomly) of 1 (p=0.1) and a significance score (likelihood of obtaining a higher difference for random assignment of values to treatment- and control groups) of 0.5 (p=0.32). Hence, the data obtained indicates the differences in kinase activities, always comparing control to inflamed skin. To detect changes caused by the different disease models, samples from diseased mice were compared to the respective negative controls (NR IgG vs. Solvent for pEBA and pMMP, Titermax vs. Solvent for aEBA). The mean kinase statistic (calculated by averaging the difference between the signal intensities of a sample and its corresponding control sample, normalized against a pooled estimate of the standard deviation in each sample, for each peptide assigned to a specific kinase) was used for further analysis: Venn diagram analyses were performed in Venny2.0 (https://bioinfogp.cnb.csic.es/tools/venny/). The pathway ontology was performed using the STRING database (37).

### Histopathology and IF staining

Biopsies from mice, taken from perilesional skin or mucosa, were fixed in 4% Histofix Solution (Carl Roth, Karlsruhe, Germany). The 6μm-thick sections from paraffin-embedded tissues were stained with hematoxylin and eosin (H&E) according to the standard protocols. The extent of dermal cell infiltration at the microscopic level was semi-quantified by an investigator unaware of the applied treatments. To detect tissue-bound IgG depositions at the DEJ, direct IF microscopy was performed on perilesional skin biopsies as detailed elsewhere (26).

### Statistical analysis

GraphPad Prism (Version 8, GraphPad Software, San Diego, CA, USA) was used for statistical analysis of the data. All animal data (EBA score over time) are presented as mean ± standard error of the mean (SEM), all other data are presented as Tukey Box and Whisker blots (showing median and the 25-75 percentiles and all “outlying” points individually). Shapiro-Wilk test was performed to examine normal distribution of data. For comparison of means and P-value determination of two groups, unpaired t test or Mann-Whitney-U test was used. For comparison of means and P-value determination of more groups, Kruskal Wallis test with Dunn´s post test was conducted. A two-way ANOVA test was performed for comparison of means of two groups in a time frame with taking the treatment into account as independent variable. All tests were followed by Sidak’s or Dunn’s multiple comparison test and adjusted P-value <0.05 was considered statistically significant. We used the STRING database (38, 39) to build connectivity networks between kinases identified herein using PamGene and kinases known to have an impact on clinical disease manifestation in experimental pemphigoid disease, specifically EBA (18–20). Here, Indeed, most interactions were observed with PI3Kδ across all models (18). Furthermore, previous work demonstrated that PI3Kδ inhibition has therapeutic effects in experimental pemphigoid (20). Thus, we used a PI3Kδ inhibitor to address if the kinome activation signature in pemphigoid diseases correlates with the treatment outcome using a PI3Kδ-selective inhibitor. To determine a possible overlap between kinase activation, we used VENNY 2.1 (https://bioinfogp.cnb.csic.es/tools/venny/).

## Results

### Unique kinase activation signatures across different pemphigoid disease models

We first obtained skin from mice with antibody transfer-induced MMP, antibody transfer-induced EBA, and immunization-induced EBA. Samples were taken at the end of the experiments; specifically, day 12 from the antibody transfer-induced models and at week 4 after randomization from immunization-induced EBA. Skin from control mice injected with either normal rabbit IgG (antibody transfer-induced models) or mock-immunized mice (immunization-induced EBA) was taken from corresponding anatomical sites at the same time points. As PI3K activation does not translate into substrate phosphorylation (40), its activation cannot be detected using multiplex kinase activity profiling based on the detection of substrate phosphorylation by kinases. We, hence, added PI3Kδ (*Pik3cd*) to the list of activated kinases identified in each model and used STRING (39) to build connectivity networks of kinase activation in the skin of mice with experimental pemphigoid disease.

**Figure 1:**
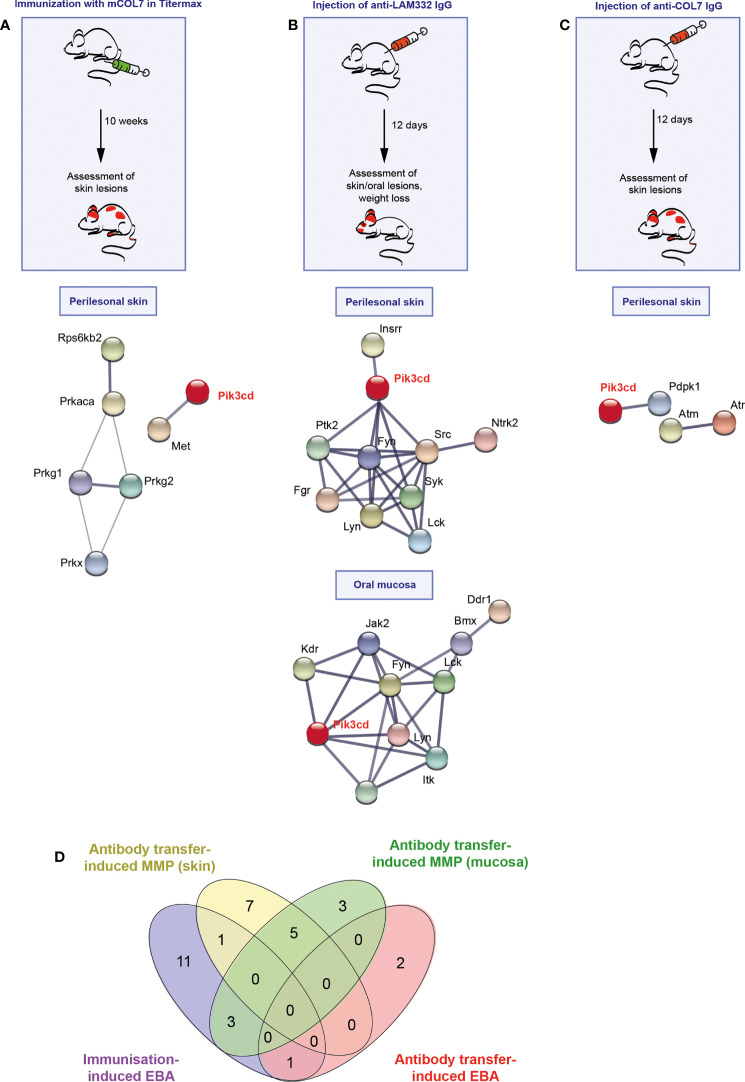
Different kinase pathways are activated in experimental models of pemphigoid diseases (PD). **(A)** For induction of immunization-induced epidermolysis bullosa acquisita (EBA), B6.s mice were immunized with 120 μg of vWFA2 domain of mCOL7 emulsified (1:1) in Titermax™ and lesional skin was taken for kinome analysis in comparison to skin of mice treated with Titermax™ only. Hence, the data shown indicates differences between the samples. **(B)** To induce experimental mucous membrane pemphigoid (MMP), C57BL/6J mice were injected every other day with 6 mg of rabbit anti-mLAMα3 IgG or NR IgG for a total of 12 days. **(C)** Antibody transfer-induced EBA was induced by six injections of 3 mg rabbit anti-mCOL7 IgG or (normal rabbit) NR-IgG for a total of 12 days. In all three models, treatment regimens and STRING database analysis of kinases that are modulated are depicted here for analysis of perilesional skin and (MMP model only) oral mucosa. STING interaction modules are shown at the medium confidence level. Thickness of the lines indicates the strength of the predicted interaction. Colors of kinases are randomly selected. Differently activated kinases that did not have any connections to other kinases were excluded from the networks. n=3/model and group. **(D)** Venn diagram of overlapping kinase profiles in the respective PD models.

In all pemphigoid disease models, differential kinase activation following disease induction was noted. The greatest number of differently activated kinases was observed in the skin of mice with immunization-induced EBA (n=17), followed by the antibody transfer-induced MMP (n=13). Whereas only three kinases were differently activated in the skin of mice with antibody transfer-induced EBA (**Figure 1**, Raw Data Table in the supplement to this article). In immunization-induced EBA, cGMP-/cAMP- dependent protein kinases dominated the network. PI3Kδ was integrated in the network through interactions with hepatocyte growth factor receptor (*Met*, a receptor tyrosine kinase, **Figure 1A**), which are both activated after FcgR stimulation in neutrophils. The kinase activation signature in antibody transfer-induced MMP centered around PI3Kδ, Syk and the Src kinase family (**Figure 1B**). By contrast, PI3Kδ only interacted with pyruvate dehydrogenase kinase isozyme 1 (*Pdpk1*) in the connectivity network of activated kinases in antibody transfer-induced EBA (**Figure 1C**). When investigating for an overlap among the three models, we found that the kinase activation signatures are distinct across the three mouse models of pemphigoid diseases (**Figure 1D**): More specifically, only 2 kinases showed an overlap: Calcium/calmodulin-dependent protein kinase type IV (*Camk4*) between antibody transfer-induced MMP and immunization-induced EBA, and serine/threonine-protein kinase ATR (*Atr*) shared between antibody transfer-induced and immunization-induced EBA.

### The PI3Kδ-selective inhibitor parsaclisib impairs immune complex-induced neutrophil activation *in vitro*

**Figure 2:**
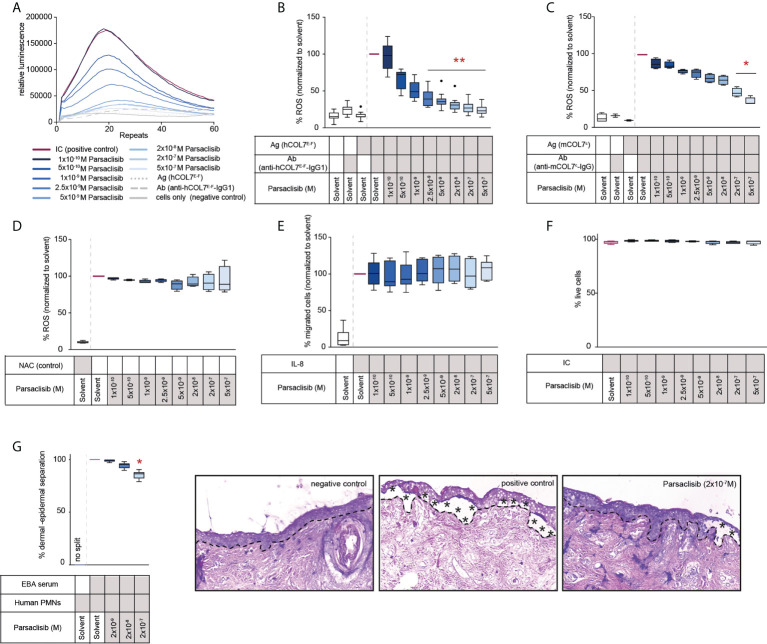
Parsaclisib impairs immune complex-induced neutrophil activation *in vitro*. Human polymorphonuclear leukocytes (PMNs) were isolated from healthy blood donors and activated by immobilized immune complexes (IC) consisting of recombinant human COL7 (hCOL7E-F) and anti-hCOL7E-F IgG1 in the presence or absence of parsaclisib at an 8-fold concentration range. Relative reactive oxygen species (ROS) release was detected by a luminescence-based assay. **(A)** Representative example of one donor showing the relative luminescence after IC-stimulation over the time. **(B)** Area under the curve (AUC, cumulative values) of luminescence, n=12. **(C)** PMNs were isolated from murine femurs and activated using immobilized ICs consisting of recombinant murine COL7 (mCOL7^C^) and anti-mCOL7^C^-IgG in the presence or absence of parsaclisib, n=4. **(D)** ROS were generated enzymatically by myeloperoxidase, catalase, and glucose oxidase, n=4. The ROS scavenger N-acetylcysteine (NAC) was used as assay control. **(E)** Chemotaxis of freshly isolated PMNs was induced by IL-8 in the presence of parsaclisib using a Boyden chamber assay. The attracted cell number during a time period of 60 minutes is shown as AUC. **(F)** Human PMNs were stimulated with immobilized ICs in the presence/absence of parsaclisib. To exclude toxicity, the amount of propidium iodide (PI)- and Annexin V-positive cells after IC stimulation was identified. **(G)** Cryosections of human skin were incubated with IgG isolated from EBA patients, followed by the addition of PMNs, isolated from healthy donors (cryosection assay). Split formation along the dermal epidermal junction (DEJ, dotted line) was analyzed as percentage of the whole DEJ. Asterisks indicate split formation. **(A-F)** Data was normalized to positive control (either IC- or IL-8-stimulated PMNs). Solvent control was always added to positive and negative controls. **(B-G)** Data are shown as Tukey’s box-and-whisker plots. ANOVA on ranks (Kruskal-Wallis) was applied followed by a Dunn´s multiple comparison test, **(B)** n=12, **(C)** n=2-4, **(D, F)** n=4, **(E)** n=5, **(G)** n=6 (for detailed information see attached raw data table), *p<0.05, **p<0.01.

We next evaluated the impact of parsaclisib on PMNs activated by immune complexes. For this purpose, human or mouse PMNs were isolated and activated by immune complexes in the presence or absence of parsaclisib at an 8-fold concentration range. In human PMNs, a significant reduction of ROS release, as a sugorrate for PMN activation, was observed at 2.5nM, with an almost complete inhibition at 0.5 μM. Compared to human PMNs, immune complex activation of murine PMNs was less sensitive to PI3Kδ inhibition. More specifically, a significant reduction of immune complex-induced ROS release was observed at the 2x10^-7^ M dose (**Figures 2A-C**). To exclude that the observed reduction of ROS is not due to any anti-oxidative properties of the compound, ROS were generated enzymatically. Here, in contrast to the treatment control, the antioxidant N-acetylcysteine (NAC), parsaclisib showed no anti-oxidative properties (**Figure 2D**). Interestingly, and differently when compared to other PI3Kδ inhibitors (20), parsaclisib had no effect on IL-8 induced PMN migration (**Figure 2E**). All effects were observed at non-toxic concentrations of parsaclisib within the same concentration range (**Figure 2F**). To validate our findings in an independent *ex vivo* model, we incubated IgG isolated from EBA patients on cryosections of human skin, followed by the addition of PMNs, isolated from healthy donors. In this cryosection assay, PMNs bind to the immune complexes located at the dermal-epidermal junction, become activated in a FcγR-mediated fashion (41), leading to ROS- and protease-dependent dermal-epidermal separation (42, 43). In cryosections incubated with EBA-IgG, PMN-dependent induction of dermal-epidermal separation was blocked by the addition of parsaclisib (**Figure 2G**).

### Parsaclisib treatment is effective in antibody transfer-induced MMP and immunization-induced EBA, but not in antibody transfer-induced EBA

**Figure 3:**
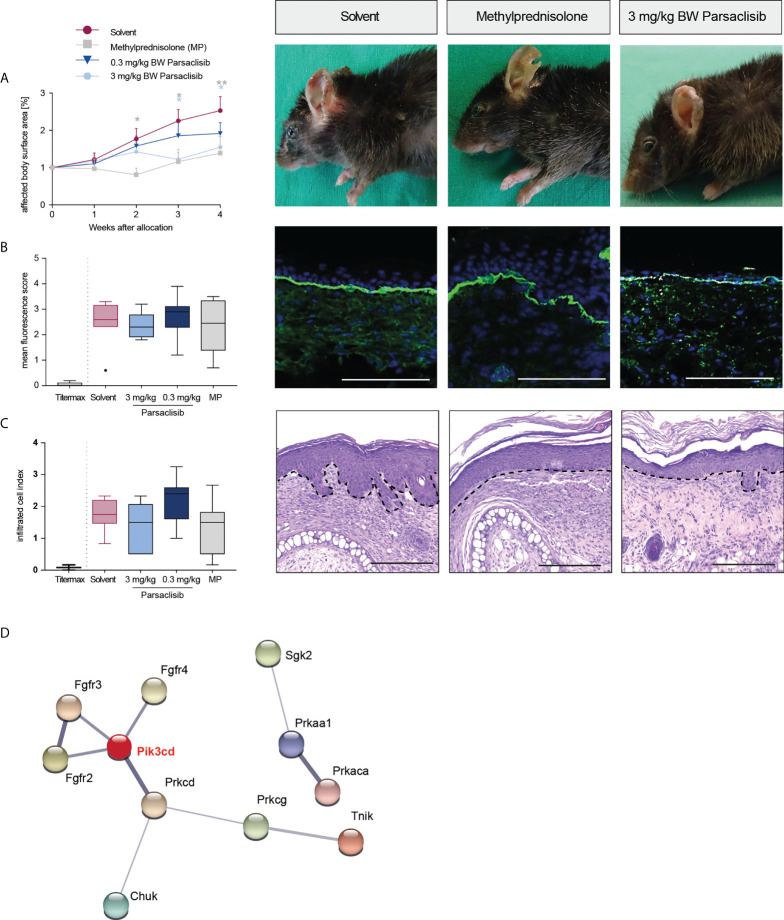
Parsaclisib improves the clinical outcome in immunization-induced EBA. B6.s mice were immunized with 120 μg of mCOL7^vWFA2^ emulsified in Titermax™. If 2% or more of the body surface area was covered with lesions in individual mice, these animals were treated with different concentrations of parsaclisib or methylprednisolone (MP), n=9/group. **(A)** Relative magnitude of affected body surface area in relation to the time of allocation over the time and representative clinical images of the mice 4 weeks after allocation. Data are shown as mean ± SD. The representative clinical images for the indicated treatments were obtained at the end of the 4-week treatment period. **(B)** To visualize IgG binding to the dermal-epidermal junction, ear skin was stained for anti-mouse IgG (green) and nuclei (DAPI, blue). Overall, no difference among the groups was noted. Representative direct IF pictures and mean fluorescent intensity of IgG binding. **(C)** Representative H&E-stained ear skin at the end of the experiment. The cumulative histological score index (amount of skin infiltration, epidermal thickening and split formation at the DEJ) was analyzed in lesional back skin. **(B-C)**, data are shown as Tukey’s box-and-whisker plots. ANOVA on ranks (Kruskal-Wallis) was applied followed by a Dunn´s multiple comparison test. **(D)** PamGene kinome analysis of lesional skin treated with solvent vs. 3 mg/ml parsaclisib (n=3/group) was performed. STRING database analysis of kinases that are modulated by parsaclisib; contrasting kinase activation in lesional skin from solvent- to parsaclisib-treated mice. STING interaction modules are shown at the medium confidence level. Thickness of the lines indicates the strength of the predicted interaction. Colors of kinases are randomly selected. Differently activated kinases that did not have any connections to other kinases were excluded from the networks. *p<0.05, **p<0.01, scale bar=100 µm.

**Figure 4:**
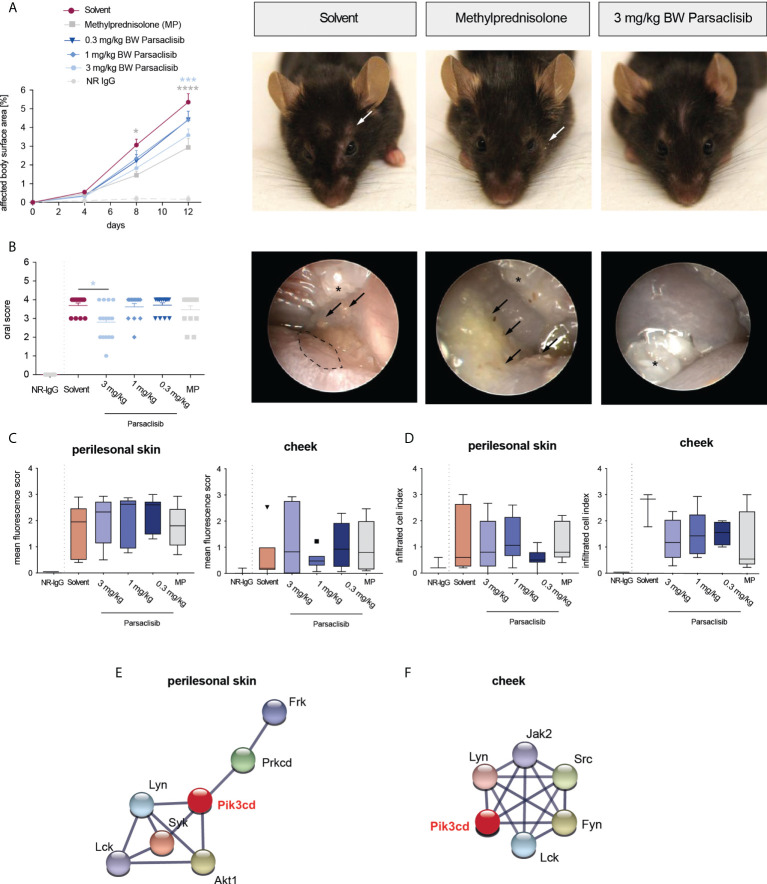
Parsaclisib improves the clinical outcome in antibody transfer-induced MMP. C57BL/6J mice were injected every other day with 6 mg of rabbit anti-mLAMα3 IgG (n=15/group) or normal rabbit (NR) IgG (n=6) for a total of 12 days. Administration of MP, solvent or parsaclisib (0.3, 1, or 3 mg/kg) was initiated on day 0 of the experiment and continued until day 11. **(A)** Percentage of affected body surface area was determined at days, 0, 4, 8 and 12. Representative clinical images of the mice at day 12. Data are shown as mean ± SD **(B)** High-resolution endoscopy of mouth oral cavity (i.e., pharyngeal mucosa, tongue, and right/left buccal) was conducted to determine the extent of oral lesions at day 12. Data are shown as scatter blot, including mean ± SEM. Representative pictures of oral cavity. Arrows indicate mucosal lesions, asterisks indicate teeth. PamGene kinome analysis of lesional skin treated with solvent vs. 3 mg/ml parsaclisib (n=3). **(C)** The mean fluorescence intensity of IgG binding to the dermal-epidermal junction was analyzed in ears or cheek (oral mucosa), respectively at the final end point. **(D)** The cumulative histological score index (amount of skin infiltration, epidermal thickening and split formation at the DEJ) of ears, or mucosa was analyzed at day 12. **(C, D)** Data are shown as Tukey’s box-and-whisker plots. ANOVA on ranks (Kruskal-Wallis) was applied followed by a Dunn´s multiple comparison test. String database analysis of kinases that are modulated by parsaclisib in **(E)** perilesional skin and **(F)** mucosa; contrasting kinase activation from solvent- to parsaclisib-treated mice. STING interaction modules are shown at the medium confidence level. Thickness of the lines indicates the strength of the predicted interaction. Colors of kinases are randomly selected. Differently activated kinases that did not have any connections to other kinases were excluded from the networks. *p<0.05, ***p<0.001, ****p>0.0001.

**Figure 5:**
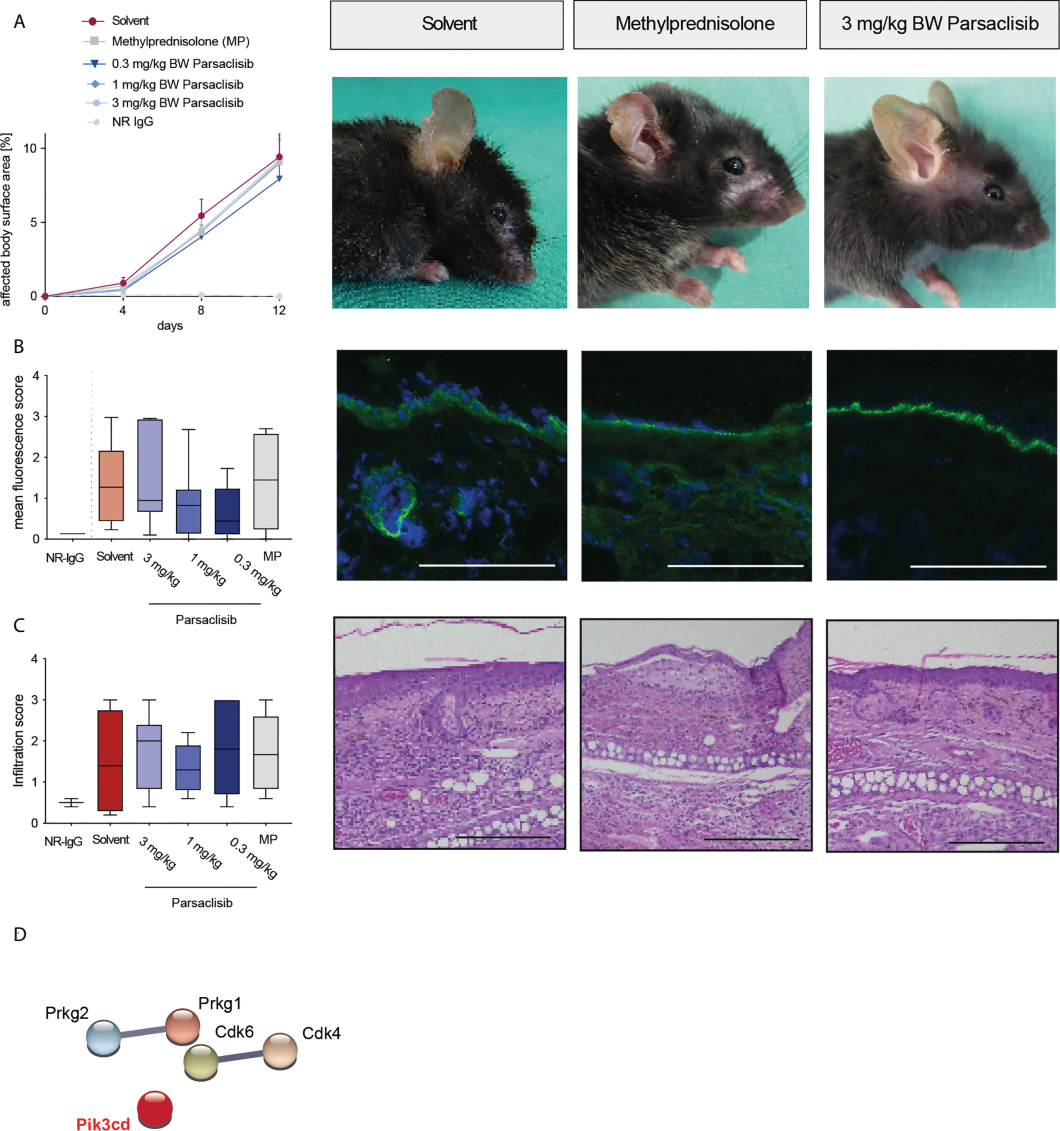
Parsaclisib does not affect antibody transfer-induced EBA. C57BL/6J mice were injected every other day with rabbit anti-mCOL^vWFA2^ IgG (n=17/group) or normal rabbit (NR) IgG (n=5) for a total of 12 days. Administration of methylprednisolone (MP), solvent or parsaclisib (0.3, 1, or 3 mg/kg) was initiated on day 0 of the experiment and continued until day 11. **(A)** Percentage of affected body surface area was measured at days, 0, 4, 8 and 12. Representative clinical images of the mice at day 12. Data are shown as mean ± SD. **(B)** To visualize IgG binding to the dermal-epidermal junction, ear skin was stained for anti-mouse IgG (green) and nuclei (DAPI, blue). Representative direct IF pictures and mean fluorescent intensity of IgG binding. **(C)** Representative H&E-stained ear skin at the end of the experiment. The cumulative histological score index (amount of skin infiltration, epidermal thickening and split formation at the DEJ) was analyzed in lesional back skin. **(B, C)**, data are shown as Tukey’s box-and-whisker plots. ANOVA on ranks (Kruskal-Wallis) was applied followed by a Dunn´s multiple comparison test. **(D)** PamGene kinome analysis of lesional skin treated with solvent or 3 mg/ml parsaclisib (n=3), respectively. String database analysis of kinases that are modulated by parsaclisib; contrasting kinase activation in lesional skin from solvent- to parsaclisib-treated mice. STRING interaction modules are shown at the medium confidence level. Thickness of the lines indicates the strength of the predicted interaction. Colors of kinases are randomly selected. Differently activated kinases that did not have any connections to other kinases were excluded from the networks, Scale bar=100 µm.

Having established that PI3Kδ is integrated in the kinome activation signature of lesional skin from mice with antibody transfer-induced MMP and immunization-induced EBA and having demonstrated that parsaclisib impairs a key pathogenic pathway in autoantibody-induced tissue pathology in pemphigoid diseases, we next evaluated the impact of parsaclisib treatment on the clinical manifestation of experimental pemphigoid. In immunization-induced EBA, parsaclisib was administered in therapeutic settings: Mice were immunized with COL7 for induction of EBA. If, in an individual mouse, 2% or more of the body surface area were affected by EBA skin lesions, it was randomized to one of the treatments. In solvent-treated mice, clinical disease worsened over 2-fold during the 4-week treatment period. By contrast, parsaclisib significantly abolished disease progression. We also included methylprednisolone as an active comparator (44). The effects of methylprednisolone at 20 mg/kg and parsaclisib at 3 mg/kg were comparable (**Figure 3**). In lesional skin from all groups, a similar degree of dermal leukocyte infiltration was observed. Likewise, a similar IgG- deposition at the dermal-epidermal junction, and comparable serum levels of COL7-specific and total IgG were noted in all treatment groups (**Figure 3**, **Supplement Figure 1**). We then determined kinase activity in lesional skin from mice treated with parsaclisib to solvent-treated mice at the end of the experiment. Like above (**Figure 1**), we included PI3Kδ to the list of differently activated kinases between the 2 groups when analyzing the data using STRING database (**Figure 3D**). In antibody transfer-induced MMP (**Figure 4**) and EBA (**Figure 5**), treatments were started when pathogenic IgG was administered for the first time and was maintained throughout the 12-day observation period. In line with the findings from immunization-induced EBA, parsaclisib (3 mg/kg) or methylprednisolone (20 mg/kg) impaired the induction of skin lesions in antibody transfer-induced MMP (**Figure 4A**). By contrast, neither MP, nor parsaclisib had an impact on clinical disease manifestation in antibody transfer-induced EBA (**Figure 5A**). In experimental MMP, involvement of the oral mucosa is frequently observed. Interestingly, parsaclisib (3 mg/kg), but not methylprednisolone (20 mg/kg) significantly reduced oral blistering and erosions (**Figure 4B**). In antibody transfer-induced MMP (**Supplement Figure 2**) or EBA (**Supplement Figure 3**), no changes in circulating specific and total IgG were noted among the treatment groups. Other secondary endpoints in all models included analysis of cytokine and leukocyte subset expression in the skin (*Cxcl1, IL17a, Tnf, Ly6g, Csf2, Cd3e, Il10, Itgam*) by RT-PCR, and determination of cytokine concentrations in the serum (IL-1β, IL-4, IL-1α, IFN-γ, TNF-α, CXCL1, IL-10, IL-13, IL-17A, GM-CSF). Here, no major changes were observed, with the exception of serum cytokine expression in antibody transfer-induced MMP (**Supplement Tables 1–6**). Here, compared to solvent treated mice, parsaclisib reduced serum concentrations of IL-1β, IL-17A and GM-CSF, as well as increasing the serum concentrations of IL-1α and CXCL1. Nothing is known about the functional relevance of these cytokines in MMP. However, if considering data from EBA and BP mouse models, IL1-β, IL-17A, GM-CSF and CXCL1 have been demonstrated to promote skin inflammation, with no contribution of IL-1α (12, 45).

In immunization-induced EBA and antibody transfer-induced MMP, PI3Kδ was linked to the kinase activation signatures when compared to healthy skin (**Figure 1**), as well as when comparing lesional skin in mice treated with solvent to lesional skin of mice treated with parsaclisib (**Figures 3D**, **4C**). In antibody transfer-induced EBA, PI3Kδ showed only few connections to the network when contrasting healthy versus lesional skin, and no interactions were observed comparing lesional skin of mice treated with solvent to those that were treated with parsaclisib (**Figure 5B**). Thus, the interaction of PI3Kδ with the kinase activation signature in inflamed skin, is associated with a favorable treatment outcome using a PI3Kδ-selective inhibitor.

## Discussion

We here determined the cutaneous kinase activation signatures in three different experimental pemphigoid disease models. Interestingly, kinase activation was unique in antibody transfer-induced MMP, antibody transfer-induced EBA and immunization-induced EBA. The differences between the two models based on antibody transfer is most likely due to the fact that different autoantigens, LAMα3 in MMP versus COL7^vWFA2^ in EBA, are targeted by the autoantibodies. Furthermore, B6 mice were used in the antibody transfer models, while the MHC-congenic B6.s strain was used for immunization-induced EBA. All other experimental conditions, i.e., animal facility, time of experiment and experimenters, were similar. This finding underscores that, also on a molecular level, pathways associated with disease pathogenesis are different between MMP and EBA. The differences in cutaneous kinase activation observed between antibody transfer- and immunization-induced EBA were rather unexpected. However, given their functional validation, i.e., different treatment outcomes after PI3Kδ inhibition, underscores their validity. The difference in the “age” of the lesions most likely contributes to the observed difference: Samples from antibody transfer-induced EBA were obtained 12 days after the first pathogenic IgG injection. Here, first lesions appeared on day 4. Hence, the cutaneous kinase signature represents early disease development. By contrast, skin samples from immunization-induced EBA were obtained at week 4 after allocation into treatment groups. Given that skin lesions develop 3-8 weeks after immunization (35), the kinome signature observed herein most likely reflects kinase activation profiles of chronic EBA skin lesions. This may be a limitation of our study. We, however, believe that the correlation of kinome signatures with treatment outcomes underscores the overall validity of the findings.

The treatment with the PI3Kδ-selective inhibitor parsaclisib showed a differential response that correlated with the integration of PI3Kδ into the cutaneous kinome activity. In those experimental models that revealed an integration of PI3Kδ into the kinome activation network, i.e., antibody transfer-induced MMP and immunization-induced EBA, treatment with parsaclisib impaired disease induction or progression of already established disease. These findings independently validate previous findings using another PI3Kδ inhibitor (LAS191954) in immunization-induced EBA (20). The potentially clinically most relevant findings are the observations in the MMP mouse model: Here, both methylprednisolone and parsaclisib partially blocked induction of skin lesions. Mucosal (oral) lesions were, however, amendable to parsaclisib but not methylprednisolone treatment. Given that MMP is still difficult to treat, with the risk of blindness in ocular involvement (46), PI3Kδ inhibition may potentially improve mucosal outcomes in MMP. Of note, parsaclisib did not have an impact on clinical disease manifestation in antibody transfer-induced EBA. The correlation of PI3Kδ integration of the cutaneous kinome signature with treatment outcomes indicates that determination of kinase activity may be useful to predict treatment outcomes in patients. While no signal transduction inhibitors are approved for pemphigoid diseases, this could be translated in patients with psoriasis and atopic dermatitis, where several small molecule kinase inhibitors have recently been licensed (47, 48).

In previous studies the PI3Kδ inhibitor LAS191954 slightly, but significantly, impaired induction of EBA in the same model (20). These differences may be due to additional, uncharacterized selectivity of the inhibitors. This assumption also stems from the observation of a unique impact of these two inhibitors regarding IL-8 induced PMN migration: LAS191954 reduced IL-8 induced PMN migration, whereas parsaclisib had no effect. Furthermore, distinct *in vivo* pharmacokinetics may also contribute to the observed differences in modulating antibody transfer-induced EBA.

In conclusion, kinase activation signatures of inflamed skin, herein exemplified by pemphigoid diseases, correlate with the therapeutic outcomes following kinase inhibition, herein exemplified by a PI3Kδ inhibitor. However, in this regard our study is not without limitations because cutaneous kinase activation profiles and treatments were performed in different mouse strains. In perspective, this could be addressed in a proof-of-concept pre-clinical trial, where in immunization-induced pemphigoid disease, cutaneous kinome signatures are determined, followed by a kinase inhibitor treatment that has the best fit to the determined network of each individual.

## Data availability statement

The original contributions presented in the study are included in the article/**Supplementary Material**. Further inquiries can be directed to the corresponding author.

## Ethics statement

The studies involving human participants were reviewed and approved by Ethical committee of the Medical Faculty of the University of Lübeck. The patients/participants provided their written informed consent to participate in this study. The animal study was reviewed and approved by Ministerium für Energiewende, Landwirtschaft, Umwelt, Natur und Digitalisierung.

## Author contributions

Conceptualization: MeP, PS, ES and RL, Methodology and Investigation: SaG, SE, SP, MK, CO, MP, NG, SK, AK, KK, IOI, KI, StG, KBo, and KBi. Visualization: KBi. Supervision: MeP, KBi, PS, ES and RL. Writing – original draft: RL. Writing – review & editing: All authors. All authors contributed to the article and approved the submitted version.

## Funding

Grant support: Cluster of Excellence “Precision Medicine in Chronic Inflammation” (EXC 2167), Research Training Group “Autoimmune Pre-Disease” (GRK 2633), and Collaborative Research Center “Pathomechanisms of Antibody-mediated Autoimmunity” (SFB 1526) from the Deutsche Forschungsgemeinschaft, the Schleswig-Holstein Excellence-Chair Program from the State of Schleswig Holstein and a Research Grant from Incyte Corporation. The funder was not involved in the study design, collection, analysis, interpretation of data, the writing of this article or the decision to submit it for publication.

## Acknowledgments

We thank Claudia Kauderer, Alexandra Wobig, Daniela Rieck and Astrid Fischer for their excellent technical support.

## Conflict of interest

MeP and PS are or were employed by Incyte. ES and RL have received research funding from Incyte.

The research was conducted in the absence of any commercial or financial relationships that could be construed as a potential conflict of interest.

## Publisher’s note

All claims expressed in this article are solely those of the authors and do not necessarily represent those of their affiliated organizations, or those of the publisher, the editors and the reviewers. Any product that may be evaluated in this article, or claim that may be made by its manufacturer, is not guaranteed or endorsed by the publisher.
