# Corrigendum: Cutaneous kinase activity correlates with treatment outcomes following PI3K delta inhibition in mice with experimental pemphigoid diseases

**DOI:** 10.3389/fimmu.2022.1099535

**Published:** 2022-11-30

**Authors:** Saeedeh Ghorbanalipoor, Shirin Emtenani, Melissa Parker, Mayumi Kamaguchi, Colin Osterloh, Manuela Pigors, Natalie Gross, Stanislav Khil’chenko, Anika Kasprick, Sabrina Patzelt, Diana Wortmann, Ibrahim O. Ibrahim, Kentaro Izumi, Stephanie Goletz, Katharina Boch, Kathrin Kalies, Katja Bieber, Paul Smith, Enno Schmidt, Ralf J. Ludwig

**Affiliations:** ^1^ Lübeck Institute of Experimental Dermatology and Center for Research on Inflammation of the Skin, University of Lübeck, Lübeck, Germany; ^2^ Incyte Research Institute, Wilmington, DE, United States; ^3^ Department of Dermatology, University of Lübeck, Lübeck, Germany; ^4^ Institute of Anatomy, University of Lübeck, Lübeck, Germany

**Keywords:** animal model, neutrophils, autoimmunity, pemphigoid, epidermolysis bullosa acquisita, mucous membrane pemphigoid, signal transduction, phosphatidylinositol 3-kinase (P13k)

In the published article, there was an error in the author list. The equal contribution of Saeedeh Ghorbanalipoor, Shirin Emtenani as first authors and that of Enno Schmidt and Ralf J. Ludwig as last authors had not been indicated. The corrected author list appears below.

Saeedeh Ghorbanalipoor1‡, Shirin Emtenani1‡, Melissa Parker2, Mayumi Kamaguchi 1, Colin Osterloh 1, Manuela Pigors 1, Natalie Gross 1, Stanislav Khil’chenko 1, Anika Kasprick 1, Sabrina Patzelt1, Diana Wortmann1, Ibrahim O. Ibrahim1, Kentaro Izumi1, Stephanie Goletz1, Katharina Boch3, Kathrin Kalies4, Katja Bieber1, Paul Smith2†, Enno Schmidt1§ and Ralf J. Ludwig1*§

†Present Address: Paul Smith, Connect Biopharma, San Diego, CA, United States

‡These authors have contributed equally to this work

^§^These authors have contributed equally to this work

The authors apologize for this error and state that this does not change the scientific conclusions of the article in any way. The original article has been updated.

## Publisher’s note

All claims expressed in this article are solely those of the authors and do not necessarily represent those of their affiliated organizations, or those of the publisher, the editors and the reviewers. Any product that may be evaluated in this article, or claim that may be made by its manufacturer, is not guaranteed or endorsed by the publisher.

