# Supplementary material for: Cutaneous kinase activity correlates with treatment outcomes following PI3K delta inhibition in mice with experimental pemphigoid diseases
Source: Front Immunol. 2022 Sep 28;13:865241. doi: 10.3389/fimmu.2022.865241 (PMC9555174; doi:10.3389/fimmu.2022.865241)
Supplement: Supplementary file 2 [file DataSheet_2.pdf]

Supplemental data to: Cutaneous kinase activity correlates with treatment outcomes following PI3K delta inhibition in mice with experimental pemphigoid diseases

Saeedeh Ghorbanalipoor[1]*, Shirin Emtenani[1]*, Melissa Parker[2], Mayumi Kamagutchi[1], Colin Osterloh[1], Manuela Pigors[1], Natalie Gross[1], Stanislav Kilchenko[1], Sabrina Patzelt[1], Diana Wortmann[1], Ibrahim Osman[1], Kentaro Izumi[1], Stephanie Goletz[1], Katharina Boch[3], Kathrin Kalies[4], Katja Bieber[1], Paul Smith[2], Enno Schmidt[1]* and Ralf J. Ludwig[1]*

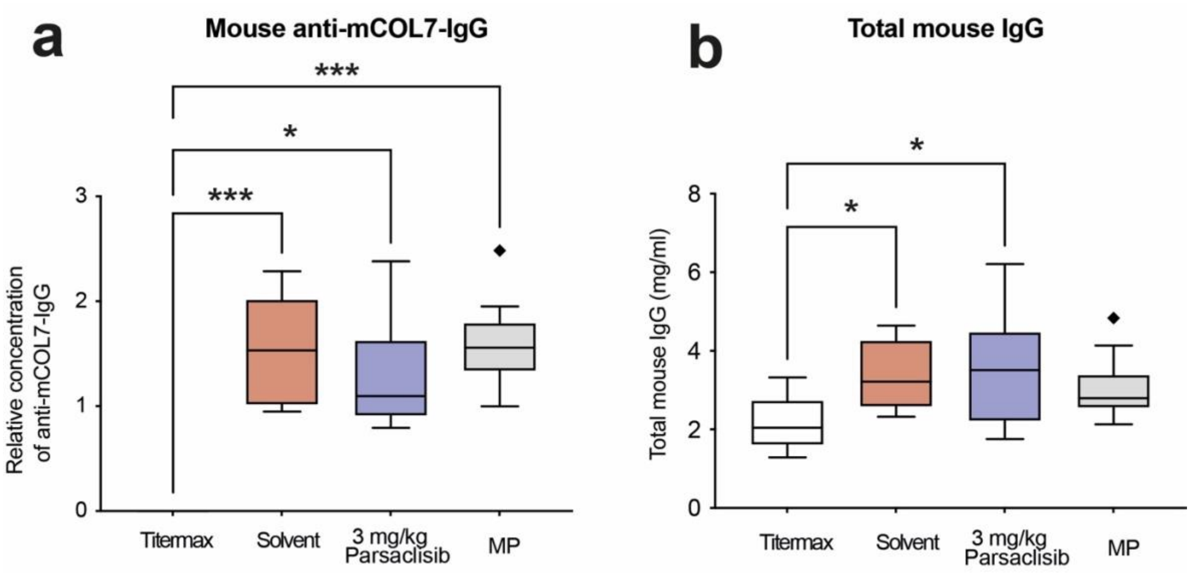

Supplement figure 1. Total and specific mouse IgG serum levels in immunization-induced EBA treated with methylprednisolone or parsaclisib. B6.s mice were immunized with 120 µg of vWFA2 domain of mCOL7 (mCOL7^{vWFA2}) emulsified in Titermax™, and serum was taken at the clinical endpoint for analysis (after a 4-week treatment period) of (**a**) specific mouse anti mCOL7-IgG and (**b**) total mouse IgG. Immunization with the antigen led to an increase in both specific and total IgG concentrations. None of the used therapeutic interventions led to significant changes in specific or total IgG concentrations. Data are shown as Tukey's box-and-whisker plots. ANOVA on ranks (Kruskal-Wallis) was applied followed by a Dunn´s multiple comparison test, n=7-10 (for detailed information see attached raw data table), *p<0.05, ***p<0.001, MP: methylprednisolon.

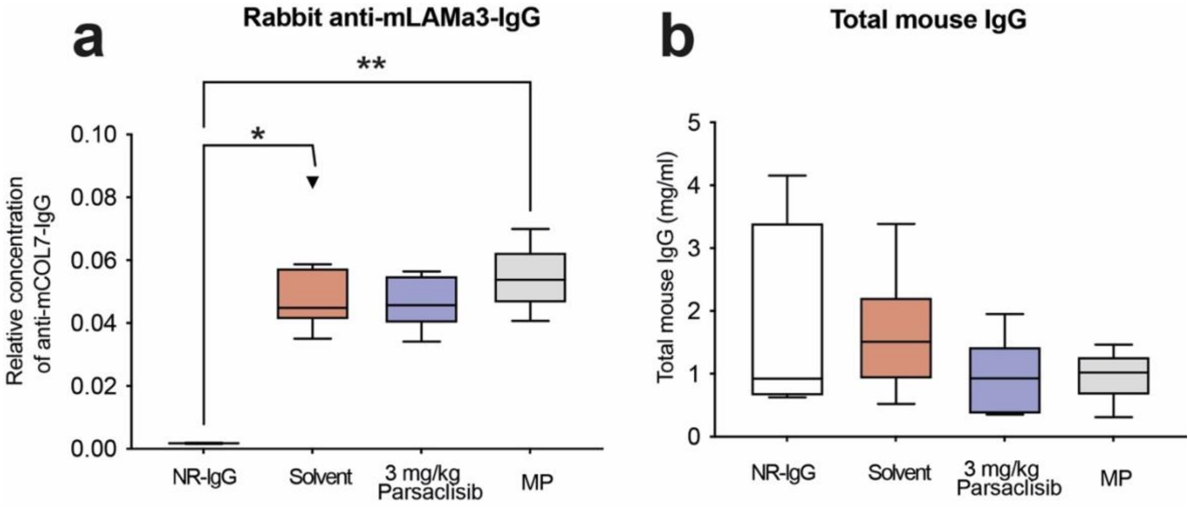

Supplement figure 2. Analysis of specific rabbit anti-mouse LAMα3 IgG and total mouse IgG serum levels during antibody transfer-induced MMP. B6 mice were injected every other day with 6 mg of rabbit anti-mLAMα3 IgG or normal rabbit (NR) IgG, and serum was taken at day 12 for analysis of (**a**) specific rabbit anti mLAMα3-IgG and (**b**) total mouse IgG.None of the applied treatments led to changes in both analytes. Data are shown as Tukey's box-and-whisker plots. ANOVA on ranks (Kruskal-Wallis) was applied followed by a Dunn´s multiple comparison test, n=4-11 (for detailed information see attached raw data table), *p<0.05, **p<0.01, MP: methylprednisolon.

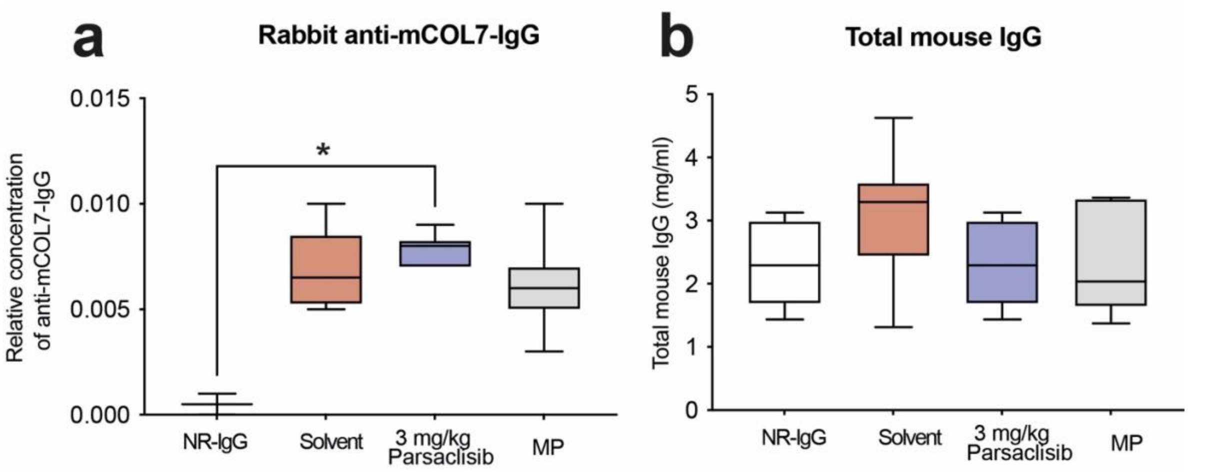

Supplement figure 3. Specific rabbit IgG and total mouse IgG serum levels during antibody transfer-induced EBA. B6 mice were injected every other day with rabbit anti-mCOL^{vWFA2}-IgG or normal rabbit (NR) IgG for a total of 12 days, and serum was taken at day 12 for analysis of (**a**) specific rabbit anti mCOL^{vWFA2}-IgG and (**b**) total mouse IgG. None of the applied treatments led to changes in specific or total IgG serum concentrations. Data are shown as Tukey's box-and-whisker plots. ANOVA on ranks (Kruskal-Wallis) was applied followed by a Dunn´s multiple comparison test, n=2-8 (for detailed information see attached raw data table), *p<0.05, MP: methylprednisolon.

Cytokine	Solvent	3 mg/kg Parsaclisib (p-value to solvent)	MP (p-value to solvent)	Titermax (p-value to solvent)
IL-1β	148.27 ± 177.92	149.02 ± 227.47	127.45 ± 178.28	226.81 ± 217.89
IL-4	13.54 ± 17.12	50.44 ± 42.04	68.16 ± 59.54	73.69 ± 64.15
IL-1α	122.81 ± 56.52	179.28 ± 148.93	114.39 ± 45.15	114.61 ± 41.16
IFN-γ	128.81 ± 96.17	236.22 ± 60.13 (*)	124.04 ± 56.14	114.82 ± 50.04
TNF-α	380.27 ± 65.62	286.23 ± 193.70	369.00 ± 194.62	294.51 ± 168.75
CXCL1	482.69 ± 168.73	640.79 ± 244.27	484.04 ± 194.17	797.61 ± 355.31
IL-10	141.90 ± 178.75	141.81 ± 226.54	234.20 ± 215.02	277.38 ± 271.09
IL-13	61.14 ± 54.35	36.28 ± 47.05	63.06 ± 34.69	78.25 ± 49.51
IL-17A	68.05 ± 90.49	37.79 ± 46.68	38.44 ± 68.69	56.74 ± 97.83
GM-CSF	0.00 ± 0.00	0.00 ± 0.00	0.00 ± 0.00	36.52 ± 45.67 (*)

Supplement table 1. Analysis of inflammatory cytokines in serum of mice after parsaclisib treatment in immunization-induced EBA. B6.s mice were immunized with 120 µg of vWFA2 domain of mCOL7 ($mCOL7^{vWFA2}$) emulsified in Titermax™, and serum was taken for LEGENDplex™ cytokine analysis. Values are indicated as picograms per milliliter serum. Mann-Whitney U-test, mean (±SD), n=7-9/group (for detailed information see attached raw data table). Significant differences in cytokine concentrations compared to solvent control are indicated in grey, *p<0.05, **p<0.01, MP: methylprednisolon.

Gene	Solvent	3 mg/kg Parsaclisib	MP	Titermax only
Cxcl1	0.07 ± 0.09	0.14 ± 0.21	0.16 ± 0.14	0.01 ± 0.02
IL17a	0.00 ± 0.00	0.00 ± 0.00	0.00 ± 0.00	0.00 ± 0.00
TNF	1.11 ± 1.22	50.76 ± 90.71	13.72 ± 23.33	5.91 ± 2.52
Ly6g	0.00 ± 0.00	0.01 ± 0.01	0.00 ± 0.00	0.00 ± 0.00
Csf2	0.26 ± 0.28	2.87 ± 5.21	1.05 ± 1.84	0.86 ± 1.01
CD3e	2.45 ± 3.42	0.92 ± 1.19	0.90 ± 1.37	6.96 ± 4.64
IL10	0.00 ± 0.00	0.01 ± 0.01	0.00 ± 0.00	0.00 ± 0.00
Itgam	1.54 ± 1.96	45.52 ± 94.68	116.04 ± 214.87	4.46 ± 3.03

Supplement table 2. Analysis of mRNA in skin biopsies of mice after parsaclisib treatment in immunization-induced EBA. B6.s mice were immunized with 120 µg of vWFA2 domain of mCOL7 ($mCOL7^{vWFA2}$) emulsified in Titermax™, and perilesional skin was taken for was taken for mRNA extraction. Analysis of mRNA by qRT-PCR for the indicated markers was done relative to the housekeeping gene GAPDH using the $2^{\Delta CT}$ method. Values are indicated as 1000-times relative copy number/copy GAPDH. Mann-Whitney U-test, mean (±SD), n=2-6/group (for detailed information see attached raw data table). Significant differences in RNA concentrations compared to solvent control are indicated in grey, MP: methylprednisolon.

**Cytokine**	**Solvent**	**3 mg/kg Parsaclisib (p-value to solvent)**	**MP (p-value to solvent)**	**NR-IgG (p-value to solvent)**
IL-1β	12.25 ± 9.23	0.00 ± 0.00 (**)	19.17 ± 10.48	7.69 ± 8.91
IL-4	2.78 ± 2.32	70.12 ± 72.38	2.28 ± 0.95	3.05 ± 1.51
IL-1α	5.51 ± 4.23	311.25 ± 213.04 (**)	8.75 ± 4.93	3.97 ± 1.60
IFN-γ	2.81 ± 3.48	47.87 ± 77.50	1.22 ± 1.17	0.00 ± 0.00
TNF-α	8.33 ± 7.36	205.76 ± 286.18	0.39 ± 1.10	11.93 ± 9.31
CXCL1	50.88 ± 34.66	1024.88 ± 600.05 (**)	56.00 ± 35.76	17.86 ± 10.68
IL-10	17.37± 15.39	454.45 ± 642.59	8.82 ± 8.68	25.45 ± 17.31
IL-13	1.82 ± 1.46	118.73 ± 92.89	1.05 ± 0.53	2.155 ± 1.92
IL-17A	2.74 ± 4.79	0.44 ± 1.30 (*)	1.17 ± 1.93	0.00 ± 0.00 (*)
GM-CSF	1.41± 1.59	0.01 ± 0.04 (*)	1.67 ± 1.13	3.19 ± 1.59

Supplement table 3. Analysis of inflammatory cytokines in serum of mice after parsaclisib treatment in antibody transfer-induced MMP. B6 mice were injected every other day with 6 mg of rabbit anti-mLAMα3 IgG or normal rabbit (NR) IgG, and serum was taken for LEGENDplex™ cytokine analysis. Values are indicated as picograms per milliliter serum. Mann-Whitney U-test, mean (±SD), n=4-12/group (for detailed information see attached raw data table). Significant differences in cytokine concentrations compared to solvent control are indicated in grey, *$p<0.05$, **$p<0.01$, MP: methylprednisolon.

Gene	Solvent	3 mg/kg Parsaclisib	MP	NR-IgG
Cxcl1	0.04 ± 0.04	0.13 ± 0.25	0.03 ± 0.02	0.03 ± 0.03
IL17a	0.00 ± 0.00	0.00 ± 0.00	0.02 ± 0.03	0.03 ± 0.04
TNF	5.72 ± 12.31	0.36 ± 0.31	1.66 ± 2.10	0.02 ± 0.03
Ly6g	0.00 ± 0.00	0.00 ± 0.00	0.02 ± 0.03	0.03 ± 0.04
Csf2	0.13 ± 0.24	0.01 ± 0.01	0.02 ± 0.03	0.04 ± 0.03
CD3e	0.02 ± 0.02	0.03 ± 0.05	0.02 ± 0.02	0.08 ± 0.03
IL10	0.00 ± 0.00	0.00 ± 0.00	0.02 ± 0.03	0.03 ± 0.04
Itgam	4.78 ± 10.96	3.96 ± 6.89	0.51 ± 0.48	0.04 ± 0.02

Supplement table 4. Analysis of mRNA in skin biopsies of mice after parsaclisib treatment in antibody transfer-induced MMP. B6 mice were injected every other day with 6 mg of rabbit anti-mLAMα3 IgG or normal rabbit (NR) IgG, and perilesional skin was taken for was taken for mRNA extraction. Analysis of mRNA by qRT-PCR for the indicated markers was done relative to the housekeeping gene GAPDH using the $2^{\Delta CT}$ method. Values are indicated as 1000-times relative copy number/copy GAPDH. Mann-Whitney U-test, mean (±SD), n=2-6/group (for detailed information see attached raw data table). Significant differences are indicated in grey, MP: methylprednisolon.

**Cytokine**	**Solvent**	**3 mg/kg Parsaclisib (p-value to solvent)**	**MP (p-value to solvent)**	**NR-IgG (p-value to solvent)**
IL-1β	109.74 ± 176.83	79.94 ± 136.40	0.00 ± 0.00	0.00 ± 0.00
IL-4	52.67 ± 41.61	89.81 ± 43.99	47.66 ± 28.35	51.34 ± 38.50
IL-1α	242.17 ± 105.98	175.12 ± 79.72	185.83 ± 103.18	231.05 ± 136.92
IFN-γ	89.63 ± 34.19	112.82 ± 28.25	70.68 ± 35.25	135.25 ± 21.88
TNF-α	156.56 ± 172.83	201.28 ± 238.34	145.50 ± 196.71	200.75 ± 246.15
CXCL1	833.72 ± 327.01	900.21 ± 279.99	813.93 ± 340.56	568.00 ± 213.33
IL-10	31.00 ± 87.59	264.19 ± 235.79	72.47 ± 134.48	249.48 ± 136.09
IL-13	80.63 ± 68.55	77.28 ± 52.27	40.48 ± 28.45	59.39 ± 33.59
IL-17A	108.49 ± 123.57	87.58 ± 101.19	5.76 ± 13.42	3.72 ± 6.44
GM-CSF	28.41 ± 46.26	60.86 ± 74.51	0.00 ± 0.00	0.00 ± 0.00

Supplement table 5. Analysis of inflammatory cytokines in serum of mice after parsaclisib treatment during antibody transfer-induced EBA. B6 mice were injected every other day with rabbit anti-mCOL^{vWFA2}-IgG or normal rabbit (NR) IgG for a total of 12 days, and serum was taken for LEGENDplex™ cytokine analysis. Values are indicated as picograms per milliliter serum. Mann-Whitney U-test, mean (±SD), n=4-10/group (for detailed information see attached raw data table). Significant differences in cytokine concentrations compared to solvent control are indicated in grey, MP: methylprednisolon.

Gene	Solvent	3 mg/kg Parsaclisib	MP	NR-IgG
Cxcl1	0.62 ± 1.14	1.92 ± 5.62	2.02 ± 4.43	0.04 ± 0.03
IL17a	0.00 ± 0.00	0.09 ± 0.26	2.01 ± 4.43	0.02 ± 0.04
TNF	5.53 ± 6.80	21.74 ± 34.63	198.86 ± 368.57	6.52 ± 5.59
Ly6g	0.11 ± 0.29	0.18 ± 0.50	2.01 ± 4.43	0.02 ± 0.04
Csf2	0.34 ± 0.47	1.52 ± 2.40	205.31 ± 451.46	0.17 ± 0.14
CD3e	0.44 ± 0.40	0.13 ± 0.30	2.03 ± 4.42	0.35 ± 0.53
IL10	0.01 ± 0.03	0.02 ± 0.04	2.01 ± 4.43	0.02 ± 0.04
Itgam	9.79 ± 11.92	42.93 ± 58.83	91.42 ± 159.93	12.16 ± 17.44

Supplement table 6. Analysis of mRNA in skin biopsies of mice after parsaclisib treatment during antibody transfer-induced EBA. B6 mice were injected every other day with rabbit anti-mCOL^{vWFA2}-IgG or normal rabbit (NR) IgG for a total of 12 days,and perilesional skin was taken for was taken for mRNA extraction. Analysis of mRNA by qRT-PCR for the indicated markers was done relative to the housekeeping gene GAPDH using the $2^{\Delta CT}$ method. Values are indicated as 1000-times relative copy number/copy GAPDH. Mann-Whitney U-test, mean (±SD), n=3-8/group (for detailed information see attached raw data table). Significant differences are indicated in grey, MP: methylprednisolon.

Supplemental material and methods

Enzyme-linked immunosorbent assay for detection of circulating IgGs

Serum levels of total mouse IgG were determined by enzyme- linked immunosorbent assay (ELISA) using mouse quantification sets (Bethyl, Montgomery, Texas, USA) following manufacturer´s protocol. For detection of mouse anti-mCOL7^{vWFA}-IgG, the same kits were used following small modifications (Pipi et al., 2021, Hammers et al., 2011). In detail, each well was coated with 250 ng recombinant mCOL7^{vWFA} in coating buffer. After blocking, diluted samples were added and incubated for 60 min. Bound antibodies were detected by HRP-conjugated goat anti-mouse antibodies (Bethyl) and tetramethylbenzidine (Invitrogen, Waltham, MA, USA). The enzymatic color reaction was stopped by 2 M sulfuric acid (Carl Roth, Karlsruhe, Germany), and the change in OD was measured with a GloMax® Discover Microplate Reader photometer (Promega, Walldorf, Germany) at 450 nm. Standard reference curves were established by using the provided mouse reference sera (Bethyl). Detection of rabbit anti-mCOL7 ^{vWFA}-IgG were performed using the same coating protocol followed by detection with the rabbit quantification set (Bethyl, Montgomery, Texas, USA). Standard reference curves were established by using the provided rabbit reference serum (Bethyl).

Analysis of serum inflammatory cytokines using Legendplex™

Serum samples of mouse experiments were diluted 1:2 in assay buffer and analyzed by Legendplex (Biolegend, San Diego, USA) using custom-made panels for IL-1β. IL-4, IL-1α, IFN-y, TNF-α, CXCL1, IL-10, IL-13, IL-17A, GM-CSF as described (Bieber et al., 2017). For subsequent FACS analysis, beads were detected using a Miltenyi MACSQuant™10 for analysis (Miltenyi, Bergisch-Gladbach, Germany). Analysis for outlier was performed by Grubbs' test. Significant outliers were excluded from further analysis.

Analysis of gene expression levels

For mRNA expression analysis in mucosa and skin, 20 cryosections (12 µm) were prepared and used for RNA isolation, reverse transcription and real-time polymerase chain reaction (RT-PCR) as described previously (Bieber et al., 2017). Briefly, total RNA was isolated according to the manufacturer's protocol (innuPrep RNA Mini Kit, Analytic Jena AG). After reverse transcription, cDNA was added either to qPCR Master Mix Plus or qPCR Master Mix SYBR green Plus (Thermo Fisher Scientific Inc., Waltham, USA) and amplified using the StepOne system (Thermo Fisher Scientific Inc.). The amount of cDNA copies was normalized to the house-keeping gene GAPDH. The following TaqMan™ Gene Expression Assays (Thermo Fisher Scientific Inc.) were used for detection: *Gapdh* (Mm99999915_g1), *Cxcl1* (Mm04207460_m1), *Tnf* (Mm00443258_m1), *Csf2* (Mm00438328_m1), *Il10* (Mm01288386_m1), *Il17a* (Mm00439618_m1), *Ly6g* (Mm04934123_m1), *Cd3e* (Mm01179194_m1), *Itgam* (Mm00434455_m1). Analysis for outlier was performed by Grubbs' test. Significant outliers were excluded from further analysis.

Supplemental references

1. Pipi E, Kasprick A, Iwata H, Goletz S, Hundt JE, Sadeghi H et al. (2021). Multiple modes of action mediate the therapeutic effect of IVIg in experimental epidermolysis bullosa acquisita. J Invest Dermatol. S0022-202X(21)02481.

2. Hammers CM, Bieber K, Kalies K, Banczyk D, Ellebrecht CT, Ibrahim SM et al. (2011). Complement-Fixing Anti-Type VII Collagen Antibodies Are Induced in Th1-Polarized Lymph Nodes of Epidermolysis Bullosa Acquisita-Susceptible Mice. J Immunol. 187, 5043-5050.

3. Bieber K, Sun S, Witte M, Kasprick A, Beltsiou F, Behnen M et al. (2017). Regulatory T Cells Suppress Inflammation and Blistering in Pemphigoid Diseases. Front Immunol. 8, 1628.
